# Hand hygiene compliance in India

**DOI:** 10.1186/1753-6561-5-S6-P259

**Published:** 2011-06-29

**Authors:** D Sureshkumar, V Ramasubramanain, K Abdulghafur, V Nagvekar

**Affiliations:** 1 Infectious disease, Apollo Hospitals, Chennai, India

## Introduction / objectives

Healthcare workers (HCWs) hands are the most common vehicle for the transmission of healthcare-associated pathogens. Evidence-based guidelines for healthcare workers’ hand hygiene practices exist, but compliance with these is internationally low. Monitoring hand hygiene compliance and providing healthcare workers with feedback regarding their performance are considered integral parts of a successful hand hygiene promotion program. But in India very few studies addressed the issue of hand hygiene compliance. The main aim of the study was to assess the hand hygiene compliance among different health care workers.

## Methods

This was a cross sectional study. The infection control professionals randomly observed the compliance of hand hygiene practices of different health care workers during their routine patient care in different wards. The HCWs were unaware that they were being observed.

## Results

In the present study 340 hand hygiene activates were observed. Alcohol based hand rub (69%) was the principle mode of hand hygiene among HCWs. Nurses (37%) & Doctors (28%) were the main participants compared to other ancillary staffs. Hand hygiene compliance before and after patient/environment contact among doctors, nurses and ancillary staffs were 66%, 62% and 54% respectively. Doctors, nurses and ancillary staffs were followed hand hygiene steps orderly in 69%, 56% and 51% of the times.

## Conclusion

Hand hygiene compliance rate among doctors and nurse were high compared to ancillary staffs. Doctors followed Hand hygiene steps orderly on most of the occasions. Ancillary staffs need to be educated about hand hygiene to improve their compliance.

## Disclosure of interest

None declared.

